# Influence of safety glasses, body height and magnification on the occupational eye lens dose during pelvic vascular interventions: a phantom study

**DOI:** 10.1007/s00330-021-08231-y

**Published:** 2021-09-08

**Authors:** Alexander Gangl, Hannes Alexander Deutschmann, Rupert Horst Portugaller, Georg Stücklschweiger

**Affiliations:** 1 Department of Radiology, Division of Neuroradiology, Vascular and Interventional Radiology, Medical University Graz, Auenbruggerpatz 9, 8036 Graz, Austria; 2 Department of Medical Physics and Radiation Protection, Medical University Graz, Auenbruggerpatz 9, 8036 Graz, Austria

**Keywords:** Fluoroscopy, Eye protective devices, Thermoluminescent dosimetry, Phantoms imaging, Cataract

## Abstract

**Objective:**

By simulating a fluoroscopic-guided vascular intervention, two differently designed radiation safety glasses were compared. The impacts of changing viewing directions and body heights on the eye lens dose were evaluated. Additionally, the effect of variable magnification levels on the arising scattered radiation was determined.

**Methods:**

A phantom head, replacing the operator’s head, was positioned at different heights and rotated in steps of 20° in the horizontal plane. Thermoluminescent dosimeters (TLD), placed in the left orbit of the phantom, detected eye lens doses under protected and completely exposed conditions. In a second step, radiation dose values with increasing magnification levels were detected by RaySafe i3 dosimeters.

**Results:**

Changing eye levels and head rotations resulted in a wide range of dose reduction factors (DRF) from 1.1 to 8.5. Increasing the vertical distance between the scattering body and the protective eyewear, DRFs markedly decreased for both glasses. Significant differences between protection glasses were observed. Increasing magnification with consecutively decreasing FOV size variably reduced the dose exposure to the eye lens between 47 and 83%, respectively.

**Conclusion:**

The safety glasses in the study effectively reduced the dose exposure to the eye lens. However, the extent of the protective effect was significant depending on eye levels and head rotations. This may lead to a false sense of safety for the medical staff. In addition, the application of magnification reduced the quantity of scattering dose significantly. To ensure safe working in the Cath-lab, additional use of protective equipment and the differences in design of protective eyewear should be considered.

**Key Points:**

*• Eye lens dose changes with physical size of the interventionist and viewing direction.*

*• The use of magnification during fluoroscopic-guided interventions reduces scattered radiation.*

## Introduction

The growing number of fluoroscopic-guided interventions [1] and an increasing uncertainness about threshold doses for radiation induced cataracts [2] emphasise the importance of adequate radiation protection. On 6 February 2018, EU member states implemented new guidelines regarding the new annual dose limit for the lens of eyes (20 mSv/a, averaged over 5 years, where the maximum for 1 year must not exceed 50 mSv) for occupational fields [3]. To fulfil these provisions, radiation protection devices are increasingly used in the daily work of professions working in the area of ionising radiation. To protect eye lenses of occupationally exposed persons, the use of leaded safety glasses is recommended[4]. In the course of fluoroscopic-guided interventions, existing eye dose thresholds of medical staff may get exceeded [5]. Therefore, the protective effect of different eyewear designs is to be assessed. In consideration of recommended threshold doses (ICRP, Publication 118), not only the relative reduction of dose exposure but also the recognition of real dose values is necessary. Consequently, the impact of body height and protection devices on the absolute dose (µGy) needs to be determined. Due to the closer proximity, the body side facing the source of radiation (patient, X-ray tube) is exposed to a higher extent of scattering dose. Thus, the interventionist’s left eye receives a higher radiation dose than the right one [6]. Consequently, to minimize the impact of anatomic structures on the one hand and to point out the sole dose-reducing capabilities of protection glasses on the other hand, dose measurements were performed at the phantom head’s left eye exclusively.

Collimating the radiation field to the area of interest reduces the scattering dose for medical stuff and thus the occupational eye lens dose too [7]. For sufficient reduction of scattered radiation, beam collimation should be conducted primarily. Using image magnification also reduces the FOV size and further increases the visibility of small structures such as micro-catheters caused by a potential improved spatial resolution the imaging system provides [8]. However, influence of magnification on dose exposure to medical staff has not been assessed in the context of pelvic vascular interventions.

The aim of this study was to prospectively assess the interventionist’s lens exposure in the angiography suite considering altering parameters (body height, head alignment, eyewear design). Furthermore, the impact of increasing magnification levels on the arising scattering dose values affecting medical staff was investigated.

## Materials and methods

Since there were no test subjects (human beings, laboratory animals, tissue samples) involved in any experimental set-ups, informed consent was waived. Approval by the institutional review board was not required. To prevent any dose exposure on the present personnel, DSA-series were started outside the Cath lab.

### Angiography system

This study was conducted on a floor-mounted angiography system (Artis zee, Siemens Healthcare). This system was equipped with a 30 × 40 cm amorphous flat-panel detector. The detector was provided with a high resolution 2 k* matrix (2480 × 1920) with a pixel size of 154 µm and 16-bit digitization depth. The system operates with a high-performance angiography X-ray tube MEGALIX Cat Plus.

### Scattering body

The utilized water phantom was an acrylic water tank (25 × 25 × 15 cm) with a 1-cm-thick wall (according to ÖNORM S5214-1) [9].

In the second part of this study, a soft tissue phantom following ICRU44-standards [10] was in use (Alderson Research Laboratories).

### X-ray radiation protection glasses

To verify the impact of the glasses’ lead equivalence and their frame design, two different models (type BR 115 and type BR 126, MAVIG GmbH) were used. Type BR 115 was equipped with 0.75-mm lead equivalence protection, whereas type BR 126 reached a lead equivalence of 0.5-mm frontal and lateral. With its significantly larger lenses at the front and the additional side lenses, BR115 is clumsier than the slim designed BR126. Their different weights were 117 g and 70 g for BR115 and BR126, respectively.

### Phantom head

The interventionist’s head was simulated by the head component of a Rando Alderson phantom (Alderson Research Laboratories). It is an anthromorphic soft tissue phantom structured in nine layers following ICRU-44 standards.

### Thermoluminescent dosimeters

Thermoluminescent dosimeters (TLDs) of type TLD 100-H (LiF: Mg, Cu, P) were in use. This sort of lithium fluoride dosimeter was doped with magnesium, copper, and phosphorus. These dosimeters are characterized by a proximate tissue equivalence and high sensitivity regarding dosimetry in low-dose fields [11]. Since TLDs do not provide absolute dose values, they have to be calibrated to pre-defined dose qualities. For this study, 120 TLD 100-H-chips were calibrated to air kerma stated in the unit microgrey (µGy) previously. To gain individual correction factors for each TLD chip, a grid filled with TLD 100-H chip was positioned in a reference field of scattered radiation obtaining individual dose responses stated in nano-coulomb (nC). Next, another semiconductor dosimeter (DIADOS Diagnostic Detector T60004) providing absolute dose values given in microgrey (µGy) was undergoing the same radiation at the same position. The detected accumulated dose was used to calculate individual correction factors µGy/nC for each TLD 100-H chip. Set-ups, like acquisition mode, scattering body (water tank) and the horizontal distance between TLDs and water phantom, were equal to the conditions of the test trail.

### RaySafe i3 real-time radiation dosimeter

The real-time dosimetry system RaySafe i3 (Unfors RaySafe AB) was an electronic dosimeter system that was especially developed for the detection and visualisation of occurring scattered radiation in the angiography suite. The RaySafe i3 personal dose meter (PDM) communicated with a real-time screen displaying the accumulated dose and the current dose rate. PDM was equipped with four semiconductor silicon diode sensors. The dosimeters were calibrated to the Personal Dose Equivalent Hp(10).

### Dose reduction factor

The dose reduction factor (DRF) is the ratio of the dose of the unprotected and protected lens of eye. At all test set-ups (eye levels and head rotations), eye lens doses were detected under protected and completely exposed conditions. These measuring results were used to calculate the DRFs for protection glasses at all eye levels including each head rotation. In addition, a DRF value (mDRF) averaged of all head rotations was determined. To be able to quantify the impact of magnification mode on the eye lens dose, DRFs were used. Therefore, dose values referring to FOV 48 were defined as unprotected set-up.

### Statistical analysis

The interpretation of data was performed using IBM SPSS Statistics 25 (IBM Inc.) and Microsoft Excel 2019 (Microsoft Corporation). A two-way analysis of variance (ANOVA test) was applied to determine statistically relevant changes regarding the performance of safety glasses. *p*-values less than 0.05 were considered being statistically significant.

### Experimental setup

**Fig. 1:**
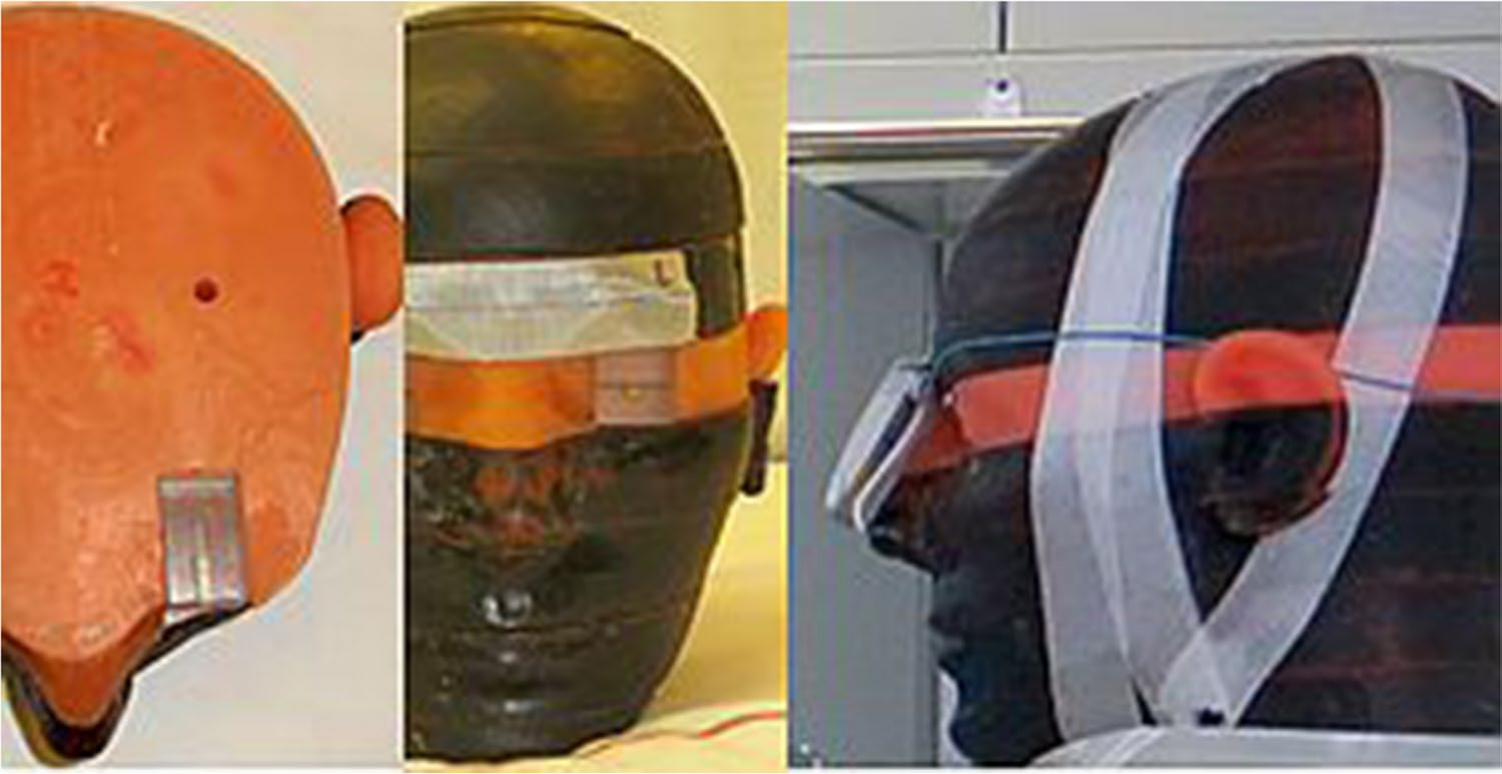
Positioning of dosimeters (TLDs, RaySafe i3) at the phantom head

**Fig. 2:**
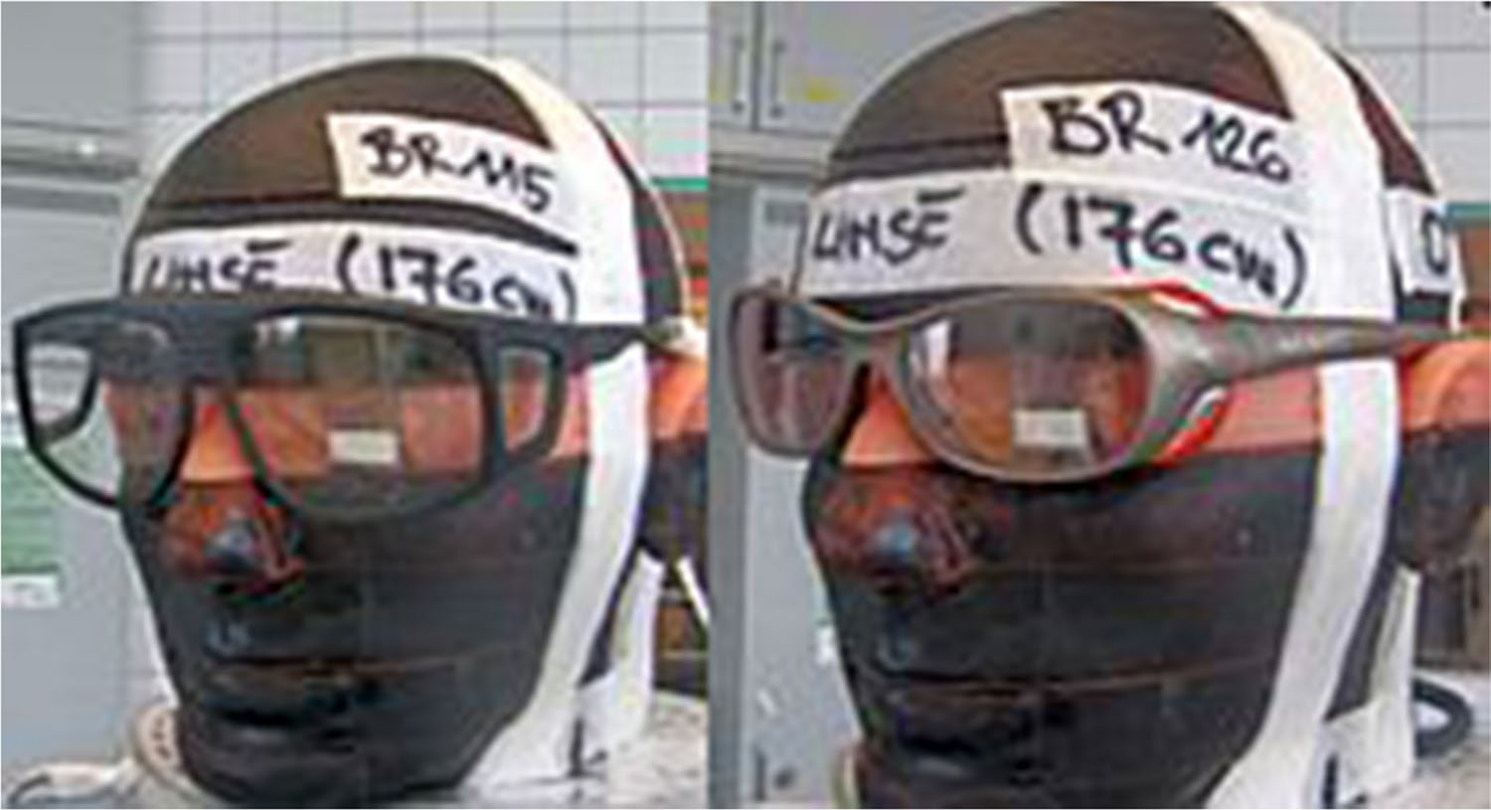
Safety glass models (left: Type BR 115, right: Type BR 126)

The water phantom was placed on the examination table of the angiography system with a fixed object height of 100 cm above the floor referring to the centre of the scattering body. The centre of the water phantom was aligned in accordance to the central beam of the X-ray tube. For each test run, an acrylic bracket was filled with calibrated TLD 100-H chips and positioned in the phantom head’s left orbit (Fig. 1). To determine the effect of body height on the radiation exposure to the lens of eye, the eye level was modified (100 cm, 157 cm and 176 cm above the floor). In order to draw comparisons between different eye levels, measuring height of 100 cm was the defined baseline. For all test runs, the horizontal distance from TLDs to the water phantom was 70 cm constantly. X-ray safety glasses of type BR115 and BR126 were positioned on the phantom head adequately (Fig. 2).

An organ-specific DSA acquisition mode (63.8 kV, 351.1 mA, 42.3 ms/image) with a predefined frame rate (6 frames/s) over 20 s was set. Automatic beam filtering (0.3 mm Cu filter) and the focal spot adjustment were pre-set by default and remained unchanged. For safety reasons, DSA runs were started outside the intervention room.

**Fig. 3:**
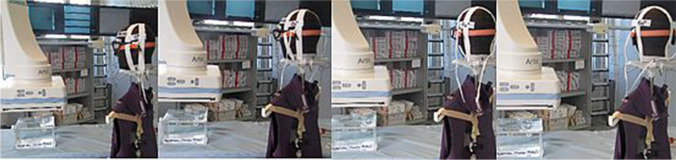
Experimental set-up: Generating eye dose values under protected conditions considering varying viewing directions (0°, 20°, 40°, 60°)

To determine the entire protective capacities of both protection glasses, the head position was turned horizontally in steps of 20° to the right (0°, 20°, 40°, 60°) increasingly oriented to the examination screen (Fig. 3). For precise alignment of all head rotations, the phantom head was positioned on a 360° protractor. This procedure was conducted at all eye levels.

In a second trial, RaySafe i3 PDM was placed at the nasal root of the dummy for getting representative eye dose values over both sides (Fig. 1). The experimental setup was the same as the previous one with regard to an eye level of 176 cm and 0° head rotation. The pelvic DSA-program was set to 7.5 frames/s over 40 s for each magnification mode. Radiation dose was detected by RaySafe i3 dosimeter continuously. Three magnification modes represented by their FOV-diagonals (48 cm, 32 cm, 11 cm) were in use. FOV-48 was defined the baseline for further analysis.

## Results

For the assessment of the efficacy of radiation protection devices, the DRF is routinely used [1, 12, 13]. The DRF is the ratio of the dose of the unprotected and protected lens of eye.

In the first test run, scattering body and eye lens were placed at the same height. This set-up delivered convincing results of both types of protection glasses. Depending on the set-up, altering dimensions of DRFs relating to type 115 and type 126 were determined. DRFs ranged from 4.8 to 8.5 for type 115 and from 2.9 to 4.3 assigning to type 126..

Altering experimental set-ups resulted in varying differences between minima and maxima of DRF values and point out the effects of viewing directions and frame designs over all head rotations (Table 1).

**Table 1:** Dose reduction factors referring to changing viewing directions, eye levels and protective eyeglass models

Eye level	Head rotation (°)	Dose reduction factor				Range
		0	20	40	60	
100	Type 115	6.9	8.0	6.0	3.9	4.0
	Type 126	3.3	4.0	2.8	2.4	1.6
157	Type 115	4.2	2.0	2.1	2.3	2.1
	Type 126	1.3	1.3	1.3	1.0	0.3
176	Type 115	2.0	1.3	1.4	1.2	0.9
	Type 126	1.5	1.1	1.3	1.1	0.4

After instating more realistic conditions by increasing height difference between the scattering body and the measuring points, determined DRFs changed in a large extent compared to the initial set-up.

Referring to interventionists of smaller stature (eye level: 157 cm), the performance of safety glasses differed significantly in comparison to the previous test series. Type 115 achieved a range of DRFs from 2.3 to 4.2 (mDRF: 3.2), respectively. Type 126, starting at lower protective effect, achieved DRFs from 1.02 to 1.3 (mDRF: 1.2) respectively. In consideration of the large spectrum of DRF-values, type 115 still showed high sensitivity to altering head alignments.

By increasing the eye level to 176 cm, the mean DRFs decreased to 1.5 (type 115; range 1.16–2.03) and 1.3 (type 126; range 1.06–1.48), respectively.

The analysis of DRFs as a function of height revealed a decreasing protective performance with increasing vertical distance between scattering body and eye lens. However, considerable differences between protection glasses of type 115 and type 126 could be discerned over all test sequences. Especially, set-ups with an eye level of 100 cm and 157 cm for type 115 achieved a better protective performance over the entire range of viewing directions. In terms of the highest eye level (176 cm), there was no significant difference (*p* = 0.729) between both types of glasses.

Table 2 displays a large range of eye dose values detected under consistent X-ray parameters but altering eye levels and protection devices. Due to the short distance to the scattering body and also because of high DRFs, maximum (683.4 µGy) and minimum (80.7 µGy) of all eye dose values were detected at 100 cm height without eye protection; eye dose values dropped almost linearly when the measuring level grew, resulting in dose decreases of 45% (157 cm height) and 62% (176 cm height), respectively. In contrast, eye dose values measured for different safety glasses at different eye levels did not decrease in the same manner.

**Table 2:** Absolute dose values considering changing eye levels, protective eyeglass models and viewing directions

	Eye level 100 cm				Eye level 157 cm				Eye level 176 cm			
Head rotation (°)	0	20	40	60	0	20	40	60	0	20	40	60
No protection (µGy)	683	642	605	565	377	341	331	322	275	204	239	229
Type 115 (µGy)	98	81	100	143	89	85	155	142	136	159	166	196
Type 126 (µGy)	205	161	217	234	299	259	263	316	186	192	179	200

**Fig. 4:**
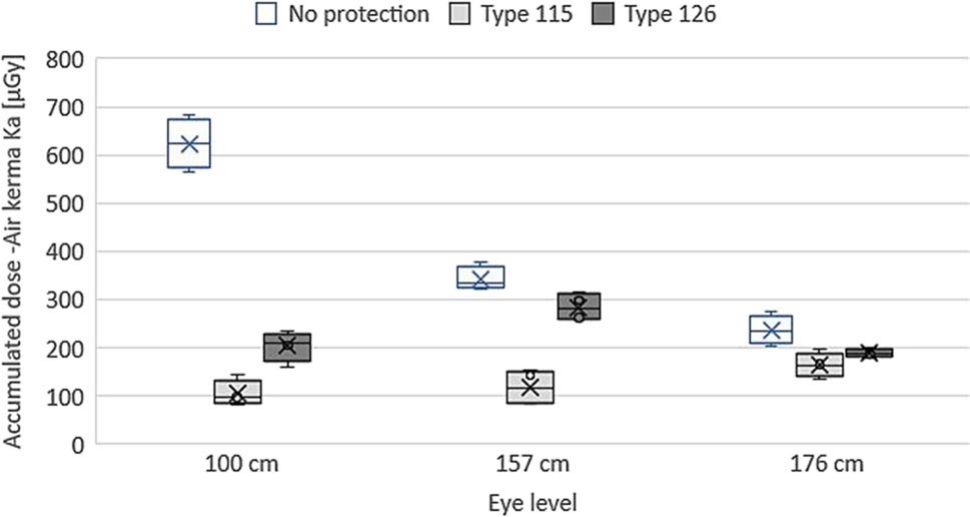
Radiation exposure to protected and unprotected eye lenses considering increasing eye levels (100, 157 and 176 cm) and head rotations (0°, 20°, 40°, 60°)

Under protected conditions, due to varying DRFs, the developments of exposure data were more irregular. Analysing the course of dose values using eyeglass model type 115, no significant difference between measuring height 100 cm (105.5 µGy) and 157 cm (117.8 µGy) was determined. However, at an eye level of 176 cm, dose exposure (164.2 µGy) was notable higher than that at measuring heights of 100 cm and 157 cm. Focusing on eyeglass model type 126, at the measuring height of 157 cm, considerable higher dose values were detected compared to those of other measuring heights (Fig. 4).

**Fig. 5:**
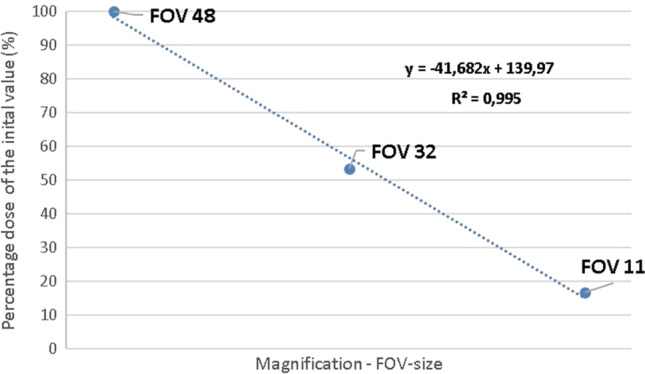
Linear regression between scattering dose and magnification level

With regard to the impact of electronic magnification on scattering dose, generated data showed a linear regression (coefficient of determination *R*^2^ = 0.995) between magnification level and scattering dose (Fig. 5). Changing the magnification level from FOV-48 to FOV-32 caused a dose drop of about 47%. By increasing the magnification to the maximum level (FOV-11), a dose reduction of about 83% in relation to the standard magnification level (FOV-48) was obtained. Transforming these figures in DRF-values results in 1.9 (FOV 32) and 6.0 (FOV 11), respectively.

According to changing magnification levels, X-ray parameters obtained from the examination protocol of the angiography system varied. Listed dose values refer to calculated air kerma at the interventional reference point (Table 3).

**Table 3:** X-ray parameters considering increasing magnification levels according to shrinking FOV sizes

FOV size	Exposure parameters					
	Tube voltage (kV)	Tube current (mA)	Radiation time per frame (ms)	Cumulative air kerma (mGy)	Dose rate (Gy/min)	Kerma-area product (µGym^2^)
FOV 48	70	288	34.3	351.711.2	2.1 ± 0.1	14614 ± 320
FOV 32	76	259	36.8	435 ± 4.0	2.4 ± 0.3	7966 ± 70.1
FOV 11	83	478	38.8	1152 ± 17.3	6.3 ± 0.1	2683 ± 35.4

## Discussion

All simulations of this study showed that parameters like the vertical gap between scattering body and eye lens, the interventionist’s head orientation and different variants in the design of protection glasses do have an effect on the occupational eye dose level. Under unprotected conditions, physicians of smaller size tend to receive higher eye lens doses than their taller colleagues do. This concurs well with previous findings [14]. However, this advantage did not persist when safety glasses were worn. Depending on the eyewear model, lower eye dose values were detected at 100 cm and 157 cm. In consideration of the initial (unprotected) dose value, an increasing eye level seems to lower the effect of safety glasses resulting in decreasing DRF values for both eyewear models. This could be explained by a lower incidence angle allowing more scatter radiation passing through the gap between safety glass and eye lens. A varying efficacy of safety glasses with changing measuring height was also stated by A. M. Koenig et al. [15]. They observed an increasing radiation dose with decreasing distance (at lower measuring height) to the scattering body. Our study can confirm these results just in part. Apart from measuring heights, also head rotations had big influences on eye dose values. Therefore, deviations in test set-up such as safety glasses, scattering bodies, measuring heights or head alignments may result in diverging findings caused by shifted photon fluence. Depending on safety glass model, we observed both increasing and also decreasing dose values at the left eye when its level increased from 157 to 176 cm.

Regardless of the eyewear design or the lead equivalent value, significant reductions of radiation exposure were attained at all measuring heights. Nevertheless, an increase of lead equivalent thickness from 0.5 to 0.75 mm did not always result in an additional protective performance immediately. A number of publications [1, 12] underline the value of a suitable design of protection glasses and degrade the weight of lead equivalence for achieving the desired protective performance [1, 16]. In addition, the effect of the viewing direction decreased with rising eye levels ending in a smaller range of DRF values for both eyewear models (Table 1).

In consideration on changing the viewing direction, the eye lens doses under protected and unprotected settings behaved contrarily. Under unprotected conditions, due to increasing distance between scattering source paired with additional anatomical barriers (bones, soft tissue), looking away from the scattering body had a positive effect on the eye lens dose. Conversely, by changing the viewing direction away from the radiation source (X-ray tube, patient) towards the examination screens, the protective extent of both types of glasses decreased. Both glasses showed their maximum protective effect when the phantom head was orientated towards the scattering body. Similar observations have been found by means of Monte Carlo simulations [17]. A lower protection efficiency of safety glasses was always observed when the rotation of the head increased.

This study has shown that depending on body height, viewing direction or eyewear model, the range of obtained DRF values was broad (1.06–8.5). These sobering but encouraging results are in line with another study simulating physician’s eye lens dose using Monte Carlo simulation presenting DRFs from 0.97 up to 6.59 depending on eyewear model and head positioning [16]. Placing safety glasses and scattering body at same level (100 cm), both eyewear models showed their best performance of all test series achieving an averaged DRF values of 5.1. These results are consistent with earlier observations [12] reporting DRFs from 5.2 to 7.6 in case of frontal impacting X-rays at the same horizontal level. Including all head rotations, the averaged DRF value dropped to 4.7.

Considering the use of magnification, this study showed that depending on the level of magnification, DRF values up to 6.0 were achieved. Increasing the magnification level reduced the quantity of scattering dose, and thus affects the major source of radiation exposition of professionals working with fluoroscopy. On the other side, obtained exposure parameters showed a tremendous increase of the cumulative air kerma (CAK) in conjunction with increasing magnification level. Since CAK is a useful indicator for patient’s peak skin dose (PSK) [18], it must be assumed that the extensive use of magnification may cause harm to patients, especially to radiation-induced skin damages. As another important issue, the kerma-area product (KAP) serves as an indicator to assess the risks of stochastic effects (e.g. cancer) and effective dose, respectively [18]. Data analysis showed that the use of magnification reduced KAP significantly ending in maximum decline of about 82%. These findings corresponded to dosimetric outcome recovered from RaySafe i3 system. Therefore, the conscious use of magnification during fluoroscopic-guided interventions can help reduce the risks of long-term damage to exposed patients. Consequently, we have to be aware of these two contradictions and need draw the necessary conclusions. Predefined threshold doses for tissue reactions have to be considered in any case. However, below these limits, a potential area for applying magnification modes exists. Especially, in critical situations where the use of protection devices is too bulky, the careful use of magnification can be a useful tool to improve the radiation protection for the patient and for the staff. Similar to our results, also Gkanatsios et al. have stated that “Magnification offers improved imaging performance at no additional patient risk provided that surface doses do not exceed the dose threshold for deterministic effects such as skin burns and epilation” [19].

Another striking argument for using magnification mode is the improvement of spatial resolution. Vendors of angiography systems often use “binning-technique” to reduce high data rates when large FOVs are in use. To reduce the amount of generated data, units of detector elements are grouped together. As a side effect, by shrinking the effective area, the spatial resolution of images declines [8]. In case of smaller FOV size, the initial data rate is smaller and no additional binning is required. Thus, to prevent the disadvantage of binning, the use of magnification mode is convincing.

For better estimation of photon energy of arising scattering radiation, the conversion factors from physical quantities of air kerma to Hp(10) were determined. Therefore, the quotients of dose responses of ionisation chambers (PM-500 (Capintec Inc.), Type 32,002 (PTW Freiburg – Physikalisch-Technische Werkstätten Dr. Pychlau GmbH)) and RaySafe i3 dosimeter given in microgrey and Hp(10), respectively, were calculated. The obtained factor of 0.915 $\frac{\mu \mathrm{Sv}}{\mu \mathrm{Gy}}$ was exemplary for a conversion coefficient in between the narrow (N) X-ray spectra 30 kV (N-30) and 40 kV (N-40). Even if the radiation spectrum of scattering radiation is not comparable to narrow (N) spectrum, an approximation of the mean energy of scattered photons was performed. The estimated energy range of scattered photons was between 24.1 and 33.1 keV.

M. Nowak et al. have shown varying energy spectra in scattered radiation fields as a function of changing measuring heights. They determined a shift of average energy of about 10% from measuring height of 96 cm to measuring height of 170 cm. Consequently, energy correction factors may vary with changing measuring height. Additionally, the position of exposed medical staff in the Cath-lab has a strong impact on radiation files. [20] With regard to our study, a slight increase of the average photon energy can be presumed. On the other hand, even if safety glasses are used, unimpeded scattered photons will reach the lens of eye. This will affect the energy spectrum of radiation behind safety glasses.

### Method errors

TLD 100-H were calibrated in a reference radiation field according to the test set-up, and the standard error of the mean was about 3%. Since any modifications (safety glasses, head rotation and measuring height) of the test set-up may influence the radiation field, an error estimation regrading TLD dosimetry is difficult. Regarding the angular response of TLDs, Pereira et al. indicated the variation of dose values to more than 50% referring to narrow X-ray spectra N-30. However, different properties of holders affect the angular response of TLDs individually [21]. RaySafe i3 detectors showed in our test series almost uniform dose values up to an angle of incidence of 60° in vertical and horizontal planes. The following specifications are listed: energy dependence < 25%, temperature dependence < 5%, dose rate uncertainty < 10% (40 µSv/h – 150 mSv/h) [22]. Therefore, we would estimate a combined error < 30%.

### Limitations of the study

In contrast to other investigations, this study was focused on radiation dose exposure of the interventionist’s left eye exclusively. Comparing our data to findings of previous studies, diverging results may be based on the individuality of test set-ups (eye wear models, scattering bodies, measuring points and head rotations). Another issue regarding the quantification of protection glasses lies in the individuality of head shapes. Even though the Rando Alderson Phantom is accepted and widely used, it represented one certain shape of head. Individual fittings were not included.

Results obtained in this study are specifically related to the set-up used and may vary using different angiography systems or dosimetry devices.

## Conclusion

The findings of this investigation confirm the value of the tested radiation protection glasses for reduction of the dose exposure to the eye lens. However, the capability to protect against ionizing radiation significantly depends on eye levels and head rotations. Increasing the lead equivalence thickness may not enhance the protective effect of safety glasses adequately but rather result in worse wearing comfort caused by the increased weight. Optimizing the shape of glasses for better individual fitting on the one hand and improvement of the protective performance against scattered radiation from below on the other hand should be considered. Nevertheless, differences between the unprotected set-up and set-ups using protection glasses were explicit and endorse the recommendation for wearing protective glasses whenever possible. The use of magnification improves the visibility of small structures and can also be an additional strategy to improve the radiation protection for the patient and the medical staff, without causing damage. Dose reduction strategies in the angiography suite should consider the differences in design of the protective eyewear and also include the use of other protective equipment like ceiling-suspended screens and under-table shields.

## Abbreviations

ANOVA: Analysis of variance
CAK: Cumulative air kerma
DRF: Dose reduction factor
DSA: Digital subtraction angiography
KAP: Kerma-area product
nC: Nano-coulomb
PSK: Peak skin dose
TLD: Thermoluminescent dosimeters

## Declarations

### Guarantor

The scientific guarantor of this publication is Univ.Prof.Dr. Hannes Deutschmann.

### Statistics and biometry

Team of the Institute for Medical Informatics, Statistics and Documentation from the Medical University of Graz kindly provided statistical advice for this manuscript.

### Methodology

• prospective

• experimental

• performed at one institution
